# Analysis of the safety of pretransplant corticosteroid therapy in patients with acute liver failure and late‐onset hepatic failure in Japan

**DOI:** 10.1002/jgh3.12508

**Published:** 2021-03-05

**Authors:** Takuro Hisanaga, Isao Hidaka, Isao Sakaida, Nobuaki Nakayama, Akio Ido, Naoya Kato, Yasuhiro Takikawa, Kazuaki Inoue, Masahito Shimizu, Takuya Genda, Shuji Terai, Hirohito Tsubouchi, Hajime Takikawa, Satoshi Mochida, Intractable Hepato‐Biliary Disease Study Group of Japan

**Affiliations:** ^1^ Department of Gastroenterology and Hepatology Yamaguchi University Graduate School of Medicine Ube Japan; ^2^ Department of Gastroenterology and Hepatology Saitama Medical University Saitama Japan; ^3^ Digestive and Lifestyle Diseases Kagoshima University Graduate School of Medical and Dental Sciences Kagoshima Japan; ^4^ Department of Gastroenterology, Graduate School of Medicine Chiba University Chiba Japan; ^5^ Division of Hepatology, Department of Internal Medicine Iwate Medical University Morioka Japan; ^6^ Department of Gastroenterology International University of Health and Welfare Narita Japan; ^7^ Department of Gastroenterology and Hematology Gifu University Graduate School of Medicine Gifu Japan; ^8^ Department of Gastroenterology and Hepatology Juntendo University Shizuoka Hospital Izunokuni Japan; ^9^ Division of Gastroenterology and Hepatology Graduate School of Medical and Dental Sciences Niigata University Niigata Japan; ^10^ Department of Gastroenterology and Hepatology Kagoshima City Hospital Kagoshima Japan; ^11^ Faculty of Medical Technology Teikyo University Tokyo Japan

**Keywords:** acute liver failure, corticosteroid, late‐onset hepatic failure, liver transplantation

## Abstract

**Background and Aim:**

In Japan, corticosteroids have been commonly used as a part of multidisciplinary therapy for patients with acute liver failure and late‐onset hepatic failure. However, there is controversy regarding the development of infections and other complications. In this study, the influence of corticosteroids on patient outcomes after liver transplantation was investigated.

**Methods:**

This study included 167 patients with acute liver failure and late‐onset hepatic failure who underwent liver transplantation between 2010 and 2015. The effects of pretransplant corticosteroid therapy on patient outcomes were evaluated using a database constructed by the subcommittee for fulminant hepatitis in the Intractable Hepato‐Biliary Diseases Study Group of Japan.

**Results:**

The subacute type and the median total bilirubin levels were higher in those receiving corticosteroids than in those not receiving corticosteroids. Although infections tended to be higher in patients receiving corticosteroids, pretransplant corticosteroid administration did not affect the survival rates. The duration from corticosteroid initiation to liver transplantation was longer in patients who developed infections. The survival rates, however, did not differ between patients with and without infections.

**Conclusions:**

Corticosteroids were administered to patients with poor prognoses. Otherwise, the overall outcome in those administered corticosteroids was not significantly different from that in those administered without corticosteroids. Although infectious complications tended to occur, they were generally controllable and nonfatal. Pretransplant corticosteroid therapy may be permissible, with regarding for infections and performed within the minimum duration.

## Graphical abstract

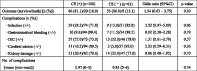

Corticosteroids are commonly used for acute liver failure in Japan. However, there is controversy regarding the development of complications. We analyzed the influence of corticosteroids on patient outcomes after liver transplantation. Although, infections tended to occur in patients receiving corticosteroids, pretransplant corticosteroid didn't affect the survival rates. The duration from corticosteroid initiation to liver transplantation was longer in patients with infections. Pretransplant corticosteroid may be a permissible treatment with caution for infections and within the minimum duration.

## Introduction

Acute liver failure is a clinical syndrome characterized by hepatic encephalopathy and a bleeding tendency due to severe liver function impairment caused by massive or submassive hepatic necrosis. Late‐onset hepatic failure (LOHF) is a disease related to acute liver failure^1^; these are both rare but life‐threatening syndromes.^2^ In Japan, a nationwide survey was carried out by the Ministry of Health, Labour, and Welfare Study Group which showed that acute liver failure and LOHF are rare diseases, as evidenced by published reports showing only 1554 acute liver failure cases and 49 LOHF cases between 2010 and 2015.^3^ Acute liver failure in coma patients, especially the subacute type, and LOHF have significantly high mortality rates,^4^ and widespread investigations into the incidence, treatment methods, and prognosis for these diseases are ongoing.^5^

Acute liver failure and LOHF can be treated with liver transplantation (LT), a standard treatment in Japan, with recent reports showing that LT was performed in 18.4% of acute, 29.2% of subacute, and 21.7% of LOHF cases.^3^ However, patients in Japan preferentially receive conservative treatment and do not undergo LT. This is mainly due to a shortage of donors, most of whom are braindead. Other reasons include the high age of patients or the high prevalence of comorbidities. The shortage of donors has been a long‐standing issue in intensive care for acute liver failure in Japan. Corticosteroid (CS) therapy is a commonly administered conservative treatment, alongside plasma exchange therapy and hemodiafiltration. CS is used to inhibit the destruction of hepatocytes and to avoid regenerative failure. Early administration is reported to be particularly effective.^6^ However, there is no clear consensus regarding the criteria for CS administration. The indication for CS administration mainly depends on the discretion of an experienced hepatologist, a protocol that has not been established by medical evidence. On the other hand, CS is also associated with increased susceptibility to infection and risk of adverse reactions, such as peptic ulcers. Because of these adverse events, pretransplant CS therapy raises concerns about impeding postoperative progress. There have been no studies published on the adverse events caused by pretransplant CS therapy on LT outcomes. Given this context, we utilized nationwide data to investigate the current status of pretransplant CS therapy in patients with acute liver failure and LOHF.

## Methods

### *Study design and patients*

We conducted a retrospective study using a database of acute liver failure and LOHF cases between 2010 and 2015, consolidated by the subcommittee for fulminant hepatitis within the Intractable Hepato‐Biliary Diseases Study Group of Japan (part of the Research Program on Rare and Intractable Diseases, Health, Labor and Welfare Sciences Research Grants). The subcommittee collects clinical information through an annual survey of cases at facilities registered in the study group. It is affiliated with the directors and fellows of The Japan Society of Hepatology, the Japanese Society of Gastroenterology, and the Japanese Association for Acute Medicine. The survey collects information on each case, including age, gender, etiology, clinical picture, treatment, and prognosis, and is conducted anonymously to protect patient information.

The requirement for informed consent was waived via the opt‐out method due to this study's retrospective design, noninvasive nature, and difficulty in obtaining consent again. Completed information about this research was disclosed on each facility's website, and the opportunity to deny participation was ensured.

All procedures implemented in studies involving human participants were performed in accordance with the ethical standards of the institutional and/or national research committee and with the tenets of the 1964 Helsinki declaration and its later amendments or comparable ethical standards. This study was approved by the Institutional Review Board (IRB), and subsequent approval was obtained from the IRB of each facility involved in the study.

### *Diagnosis of acute liver failure and LOHF*

Acute liver failure or LOHF was diagnosed based on the guidance of the 2011 Japanese Acute Hepatic Failure Study Group.^1^ Patients with a prothrombin time of 40% or less of the standardized value, or an international normalized ratio (INR) of 1.5, due to severe liver damage within 8 weeks of the onset of disease symptoms were diagnosed with acute liver failure. Furthermore, liver function prior to the current onset of liver damage should have been estimated as normal based on blood laboratory data and imaging examinations. Acute liver failure was classified as acute liver failure without hepatic coma or with hepatic coma,^1^ where no or grade I hepatic encephalopathy is present in the former, while grade II or more severe hepatic encephalopathy is found in the latter. Acute liver failure with hepatic coma was further subclassified into two disease types: acute and subacute, with grade II or more severe hepatic encephalopathy developing within 10 days or between 11 and 56 days after the onset of disease symptoms, respectively. In addition, the severity of HE was diagnosed according to the classification presented at the Inuyama Symposium in 1972. Furthermore, patients showing prothrombin time values of less than 40% of the standardized value or INRs of 1.5 or more and grade II or a more severe hepatic coma between 8 and 24 weeks since the onset of disease symptoms were diagnosed with LOHF.

With case data collected retrospectively, a diagnosis based on the revised criteria in 2011 was made for liver failure patients treated since 2010. This study used data from 2010 to 2015, reported in 2018 by a study group.^3^

### *Pretransplant* *CS* *therapy in* *LT* *patients*

We compared patients who underwent LT for acute liver failure or LOHF with and without pretransplant CS therapy. Comparisons were based on patients' clinical characteristics (age, gender, comorbidities, disease type and etiology of liver failure, and blood test values), outcomes (posttransplantation mortality), types and numbers of complications both pre‐ and posttransplantation (infection, gastrointestinal bleeding, disseminated intravascular coagulation, cerebral edema, kidney damage, etc.), and clinical course (the period from onset of disease symptoms, from CS initiation, and the development of hepatic coma grade II or more until LT) to examine the influence of CS therapy on the clinical course.

The classification of etiologies of acute liver failure and LOHF was based on our study group's criteria.^7^, ^8^ The diagnosis of autoimmune hepatitis was based on the hepatologist's decision, with reference to the diagnostic guide, in Japan in 2013, including the presence of serum antinuclear antibody or antismooth muscle antibody, high serum immunoglobulin G levels, histological features, and so on.^9^

### *Statistical analysis*

Statistical analyses were performed using the JMP Pro version 13.0.0 (SAS Institute Japan, Tokyo, Japan), and group comparisons were conducted using Fisher's exact test, the Chi‐squared test, and the Mann–Whitney *U* test. A *P*‐value <0.05 was considered statistically significant.

## Results

### *Comparison of baseline characteristics between patients who underwent* *LT, with or without pretransplant* *CS* *therapy*

Of the 170 liver transplant patients, 106 received CS therapy, 61 did not, and CS therapy status was unknown in 3 patients. In comparison with the baseline clinical characteristics, the proportion of the subacute type was significantly higher in patients who received CS therapy (69/106 patients, 65.1%) than in those who did not (17/61, 27.9%) (*P* < 0.01). In contrast, the proportion of patients with the acute type was significantly higher in the group that did not receive CS therapy (32/61, 52.5%) than in the group that did (26/106, 24.5%) (*P* < 0.01), and the proportion of patients without a coma was significantly higher in the group that did not receive CS therapy (9/61, 14.8%) than in the group that did (5/106, 4.7%) (*P* = 0.03). When comparing etiologies, CS therapy was frequently used in autoimmune hepatitis cases (*P* < 0.01) and infrequently in other cases (*P* < 0.05) (Table 1).

**Table 1:** Clinical characteristics of patients who underwent liver transplantation, comparing corticosteroid use

	CS (+) (*n* = 106)	CS (−) (*n* = 61)	*P*‐value
Age (years) median (range)	41 (1–67)	39 (1–68)	0.25
Gender (male/female) [*n* (%)]	33 (31.1)/73 (68.9)	25 (41.0)/36 (59.0)	0.24
Comorbidities (+/−) [*n* (%)]	42 (39.6)/64 (60.4)	24 (39.3)/37 (60.7)	0.97
Disease type [*n* (%)]			
Without coma	5 (4.7)	9 (14.8)	0.03*
Acute type	26 (24.5)	32 (52.5)	<0.01*
Subacute type	69 (65.1)	17 (27.9)	<0.01*
Late‐onset hepatic failure	6 (5.7)	3 (4.9)	0.84
Etiology [*n* (%)]			
Hepatitis A	2 (1.9)	2 (3.3)	0.58
Hepatitis B			
Transient infection	13 (12.3)	8 (13.1)	0.87
Acute exacerbation or de novo	7 (6.6)	3 (4.9)	0.65
Hepatitis C	2 (1.9)	0	0.18
Drug‐induced liver injury (allergic/toxic)	13 (12.3)/1 (0.9)	5 (8.2)/0	0.41/0.33
Autoimmune hepatitis	16 (15.1)	1 (1.6)	<0.01*
Others (e.g. circulatory disturbance)	6 (5.7)	10 (16.4)	0.03*
Indeterminate	46 (43.4)	32 (52.5)	0.26
Liver transplant donor [*n* (%)]			
Living/brain death/unknown	62 (58.5)/29 (27.4)/15 (14.2)	34 (55.7)/15 (24.6)/12 (19.7)	0.88

^*^ Statistically significant (*P* < 0.05).

CS, corticosteroid.

Regarding the results of blood tests taken on admission, total bilirubin level was significantly higher in patients receiving CS therapy (*P* < 0.05). In contrast, transaminase levels were lower in patients receiving CS, with a significant difference in aspartate aminotransferase (AST) (*P* < 0.05) (Table 2).

**Table 2:** Laboratory data of patients who underwent liver transplantation on admission comparing corticosteroid use

	CS (+) (*n* = 106)	CS (−) (*n* = 61)	*P*‐value
ALT (U/L)	594 (306, 1393)	790 (242, 2233)	0.30
AST (U/L)	547 (155, 1090)	912 (272, 2997)	0.03*
T‐Bil (mg/dL)	13.2 (7.1, 18.7)	9.1 (5.0, 15.9)	0.04*
BUN (mg/dL)	7.8 (4.1, 14.7)	7.0 (2.9, 16.6)	0.75
Cre (mg/dL)	0.58 (0.47, 0.75)	0.61 (0.40, 0.96)	0.66
NH_3_ (μg/dL)	116 (67, 168)	94 (80, 140)	0.64
PT% (%)	31.0 (23.0, 45.0)	32.0 (22.2, 37.0)	0.41
WBC (/μL)	7680 (5200, 10 000)	7465 (5715, 9388)	0.90
Plt (10^4^/μL)	12.1 (8.8, 16.6)	9.9 (7.6, 15.3)	0.27
CRP (mg/dL)	0.37 (0.20, 1.05)	0.31 (0.19, 1.82)	0.89

^*^ Statistically significant (*P* < 0.05).

Data are presented as medians and interquartile ranges.

ALT, alanine aminotransferase; AST, aspartate aminotransferase; BUN, blood urea nitrogen; CRP, C‐reactive protein; CS, corticosteroid; NH_3_, blood ammonia; Plt, platelet count; PT, prothrombin time; T‐Bil, total bilirubin; WBC, white blood cell count.

### *Comparison of outcomes between patients who underwent* *LT* *treated with or without pretransplant* *CS* *therapy*

Regarding the outcomes and complications after LT, there was no significant difference in mortality between patients who received CS therapy and those who did not (86 survived/20 deaths and 53 survived/8 deaths, respectively) (*P* = 0.39). There was also no difference in the mean number of complications (0.97 and 0.85, respectively) (*P* = 0.74). After excluding patients with unknown data, we discovered that more infections occurred in patients receiving CS therapy (29/103, 28.2%) than in those not receiving CS therapy (9/60, 15.0%); however, the difference was not statistically significant (*P* = 0.06) (Table 3). Infections were common in the respiratory system, blood (sepsis, catheter‐related, etc.), and abdomen (enteral or intraperitoneal) in descending order in both groups (data not shown). In these cases, data concerning pathogens such as viruses, bacteria, and fungi were insufficient.

**Table 3:** Outcome and complications of liver transplantation comparing corticosteroid use

	CS (+) (*n* = 106)	CS (−) (*n* = 61)	Odds ratio (95% CI)	*P*‐value
Outcome (survive/death) [*n* (%)]	86 (81.1)/20 (18.9)	53 (86.9)/8 (13.1)	1.54 (0.63–3.75)	0.39
Complications [*n* (%)] (※1)				
Infection (+/−)	29 (28.2)/74 (71.8)	9 (15.0)/51 (85.0)	2.22 (0.97–5.09)	0.06
Gastrointestinal bleeding (+/−)	10 (9.6)/94 (90.4)	7 (11.5)/54 (88.5)	0.82 (0.30–2.28)	0.79
DIC (+/−)	27 (27.0)/73 (73.0)	13 (22.0)/46 (78.0)	1.31 (0.61–2.79)	0.57
Cerebral edema (+/−)	11 (10.5)/94 (89.5)	3 (5.0)/57 (95.0)	2.23 (0.59–8.31)	0.26
Kidney damage (+/−)	21 (20.4)/82 (79.6)	14 (23.0)/47 (77.0)	0.86 (0.40–1.85)	0.70
No. of complications [mean (min–max)]	0.97 (0–5)	0.85 (0–4)		0.74

(※1) Patients without records of the occurrence of each complication were excluded.

CI, confidence interval; CS, corticosteroid; DIC, disseminated intravascular coagulation.

The clinical courses of patients with infections who received CS and those who did not were analyzed. The time between the onset of liver failure symptoms and LT was longer in patients with infections (*P* = 0.02). The duration from CS therapy to LT and from grade II hepatic encephalopathy emergence to LT was also longer in patients with infectious complications (*P* = 0.0498 and *P* < 0.01, respectively). In contrast, there was no significant difference in the median total CS dosage between patients with and without infections (3000 mg for both) (*P* = 0.56). Regarding outcomes, we also found no significant difference in mortality (*P* = 0.20) (Table 4).

**Table 4:** Clinical courses of patients who underwent liver transplantation after corticosteroid administration comparing the occurrences of infectious complications

	Infection (+) (*n* = 29)	Infection (−) (*n* = 74)	*P*‐value
Clinical courses (days) [median (IQR)]			
Onset of symptoms ~ CS admin.	16 (8, 29)	15 (7, 23)	0.47
CS admin. ~ LT	21 (12, 31)	14 (8, 20)	0.0498*
Encephalopathy ~ LT	15 (9, 24)	8 (3, 15)	<0.01*
Onset of symptoms ~ LT	38 (27, 73)	28 (17, 42)	0.02*
Total dose of CS (mg) [median (IQR)]	3000 (1699, 3652)	3000 (1000, 3573)	0.56
Outcome [*n* (%)]			
Survival/death	21 (72.4)/8 (27.6)	62 (83.8)/12 (16.2)	0.20

* Statistically significant difference (*P* < 0.05).

The dose of CS (mg): the dose was converted to methylprednisolone.

admin., administration; CS, corticosteroid; IQR, interquartile ranges; LT, liver transplantation.

## Discussion

The prognosis of acute liver failure is thought to be poor, and various treatments are used to improve survival, including plasma exchange therapy, hemodiafiltration, CS therapy, and LT.^10^ Of the conservative treatments, early administration of high‐dose CS inhibits the immune response‐mediated destruction of hepatocytes and microangiopathy, thereby preventing impairment of liver regeneration.^2^ The mechanism of the effect of CS also raises concerns about the risk of complications such as infections and gastrointestinal bleeding.^11^ Prior reports that show the efficacy of CS therapy are primarily in Japanese,^12^, ^13^ while randomized controlled trials (RCTs) and other studies conducted in the United States and Europe have found no improvement in survival rates.^14^, ^15^ Although the reason for this contradiction is unknown, there has been discussion regarding the prior results, which seem to be premature because the administration of CS was not uniform.^16^ It is unclear whether CS therapy increases the incidence of infections and gastrointestinal bleeding as only one report noted a limited increase in these complications.^17^

Registry data maintained by the Japanese Liver Transplantation Society shows that, of all LTs performed in Japan by the end of 2017, 91 of 364 braindead donor transplants and 817 of 8572 living‐donor transplants had primary acute liver failure.^18^ There is a social context behind why many transplanted livers come from living donors in Japan.^19^ In cases of acute liver failure in Japan, nonsurgical treatment, including CS therapy, is prioritized before surgical treatment. When nonsurgical treatment fails to elicit an improvement, indications for LT are evaluated as early as possible based on complications and recipient status.^20^, ^21^, ^22^ LT is performed once a suitable donor is found. However, due to complications related to CS therapy, concerns have been raised that pretransplant conservative CS therapy may influence the posttransplant clinical course.

This study is based on national survey data from major facilities in Japan that treat acute liver failure and perform liver transplants. Therefore, this study presents a national overview of the current medical practices for acute liver failure in Japan. This study showed that many patients who underwent LT received CS therapy before surgery (63% of all cases). In Japan, nonsurgical treatments, such as artificial liver support, plasma exchange therapy, and anticoagulation therapy, are used for as long as possible while awaiting potential transplantation. CS is commonly used for nonsurgical treatment. However, there is no clear consensus regarding the criteria for CS administration.

We found that CS is administered at an incredibly high frequency in subacute liver failure and LOHF cases. The data also showed that patients receiving CS therapy had high bilirubin and low transaminase levels on initial examination, a finding thought to reflect the trend characteristics of these types of liver failure. Nevertheless, even though CS were often used in patients with poor prognosis, we saw no difference in the outcome (mortality) or the number of complications between patients receiving CS therapy and those who did not. We consider this noninferiority of outcome to be clinically meaningful in terms of the difference in severity between patients with and without CS.

The only difference identified between patients with and without CS therapy that requires attention is the higher risk of infections in patients receiving CS therapy. The time from CS administration until LT was significantly longer in patients with infectious complications. These complications could be due to the extra time needed to manage infections before LT or because infections may likely occur in patients waiting longer for LT. After disease onset, infection prevention and management are essential to evaluate the CS therapy response and quickly determine when to opt for transplantation. Yasui *et al*. reported that the median time from starting CS therapy for acute liver failure to infection onset was 15 days. They demonstrated that determining the treatment response and opting for transplantation 2 weeks after initiating CS therapy is vital for preventing infections.^16^ Nevertheless, no significant differences in outcomes between patients with and without infectious complications were observed in this study, showing that infections did not worsen outcomes and thus could be controlled.

However, in this study, we are not going to certify the absolute safety of CS or make any haphazard recommendations. The primary limitation of this study was that, although data were collected on total CS dosage and administration methods, data on dose amounts were often missing, and no data were collected on continuous CS administration duration. More detailed data on total CS dosage and continuous CS administration duration from more patients are needed to perform a more detailed analysis of CS therapy effects. Furthermore, the results of this study merely depend on the retrospectively collected data. It is difficult to conduct case–control studies ethically and clinically because of the critical condition of patients with acute liver failure. The safety and efficacy of pretransplant CS therapy need to be reevaluated by worldwide/multicenter studies or using other strategies.

We conclude that CS therapy is commonly induced in patients with acute liver failure or LOHF in Japan and may be a permissible treatment for patients who require LT, assuming that CS administration duration is kept to a minimum. Furthermore, care needs to be taken to strictly prevent infections.

## Supporting information


**Appendix S1.** Supporting information.
