# Supplementary material for: Analysis of the safety of pretransplant corticosteroid therapy in patients with acute liver failure and late‐onset hepatic failure in Japan
Source: JGH Open. 2021 Mar 5;5(4):428–33. doi: 10.1002/jgh3.12508 (PMC8035437; doi:10.1002/jgh3.12508)
Supplement: Supplementary file 1 — Appendix S1. Supporting information. [file JGH3-5-428-s001.pdf]

Analysis of the safety of pretransplant corticosteroid therapy in patients with acute liver failure and late-onset hepatic failure in Japan

Supplemental text.

Corticosteroid therapy in the non-liver transplanted group

For Review Only

Introduction and Methods

The study in the main text refers to patients who received liver transplantation (LT). However, there were patients who did not undergo LT despite their eligibility, due to severe systemic conditions or complications, and consequently died. To analyze the safety of pretransplant corticosteroid therapy, we analyzed these LT-considered, but not transplanted patients, compared with or without corticosteroids. In this analysis, patients who could not undergo LT irrespective of the clinical course due to either lack of suitable donors or refusal by themselves or families were excluded. The consideration of LT for each case refers to the attending doctor's recommendation in case data. Using these non-LT patients' data, we also compared the influence of CS in the same method, as noted in the LT group.

Results and Discussions

As a result, of the 112 non-LT patients, 77 received CS therapy, and 35 did not. There were no significant differences in the patients' backgrounds between groups (Suppl. Table 1). As a result, there was no increase in non-LT cases caused by the development of complications in patients with CS. There was no significant increase in complications in patients with CS, but statistically little about infection or kidney damage than in patients without CS (Suppl. Table 2). In this result, there was no evidence that CS therapy aggravated the complications or disturbed the preconditions for LT. These results indicate that pretransplant CS therapy for acute liver failure is not a contraindication, provided that appropriate caution is exercised.

Suppl. Table 1: Clinical characteristics of patients in the non-liver transplant group, comparing corticosteroid use

	CS (+) (n=77)	CS (−) (n=35)	p-value
Age (yeas) [median (range)]	54 (1–85)	57 (26–76)	0.31
Sex (male/female) [n (%)]	39 (50.6)/38 (49.4)	23 (65.7)/12 (34.3)	0.16
Comorbidities (+/-) [n (%)]	37 (48.1)/40 (51.9)	22 (62.9)/13 (37.1)	0.16
Disease type [n (%)]			
without Coma	8 (10.4)	1 (2.9)	0.27
Acute type	29 (37.7)	15 (42.9)	0.68
Subacute type	33 (42.9)	12 (34.3)	0.42
Late-onset hepatic failure	7 (9.1)	7 (20.0)	0.13
Etiology [n (%)]			
Hepatitis A	4 (5.2)	0 (0)	0.31
Hepatitis B			
Transient infection	16 (20.8)	7 (20.0)	1.00
Acute exacerbation or de novo	11 (14.3)	6 (17.1)	0.78
Hepatitis C	0 (0)	1 (2.9)	0.31
Drug-induced liver injury(allergic/toxic)	1 (1.3)/5 (6.5)	0 (0)/5 (14.3)	1.00/0.28
Autoimmune hepatitis	9 (11.7)	1 (2.9)	0.17
Others (e.g., circulatory disturbance)	9 (11.7)	2 (5.7)	0.50
Indeterminate	22 (28.6)	13 (37.1)	0.39

* Statistically significant (p<0.05), CS: corticosteroid

For Review Only

Supple. Table 2: Outcomes & complications of the non-liver transplant group, comparing corticosteroid use

	CS (+) (n=77)	CS (－) (n=35)	Odds ratio (95% CI)	p-value
Reason of non-LT [n (%)]				
Severe systemic condition	24 (31.2)	11 (31.4)	0.99 (0.42–2.34)	0.98
Complications (e.g., infection)	20 (26.0)	9 (25.7)	1.01 (0.41–2.53)	0.98
Others or unknown	33 (43.8)	15 (42.9)		
Complications [n (%)] (※1)				
・Infection (+/-)	37 (50.0)/37 (50.0)	25 (73.5)/9 (26.5)	0.36 (0.15–0.87)	0.02*
・Gastrointestinal bleeding (+/-)	19 (25.0)/57 (75.0)	4 (12.9)/27 (87.1)	2.25 (0.70–7.26)	0.20
・DIC (+/-)	46 (62.2)/28 (37.8)	21 (63.6)/12 (36.4)	0.94 (0.40–2.20)	0.88
・Cerebral edema (+/-)	26 (36.6)/45 (63.4)	7 (23.3)/23 (76.7)	1.90 (0.72–5.03)	0.25
・Kidney damage (+/-)	41 (56.2)/32 (43.8)	27 (77.1)/8 (22.9)	0.38 (0.15–0.95)	0.03*
No. of complications [mean]	2.47	2.63		0.57

* Statistically significant (p<0.05), CS: corticosteroid

(※1) Patients without records of the occurrence of each complication were excluded.

DIC: disseminated intravascular coagulation, CI: confidence interval

For Review Only
